# Metabolic interventions improve HBV envelope-specific T-cell responses in patients with chronic hepatitis B

**DOI:** 10.1007/s12072-023-10490-4

**Published:** 2023-03-28

**Authors:** Yu-Long Fu, Shuang-Nan Zhou, Wei Hu, Jing Li, Ming-Ju Zhou, Xiao-Yu Li, You-Yuan Wang, Peng Zhang, Si-Yuan Chen, Xing Fan, Jin-Wen Song, Yan-Mei Jiao, Ruonan Xu, Ji-Yuan Zhang, Cheng Zhen, Chun-Bao Zhou, Jin-Hong Yuan, Ming Shi, Fu-Sheng Wang, Chao Zhang

**Affiliations:** 1 Savaid Medical School, University of Chinese Academy of Sciences, Beijing, China; 2 Senior Department of Infectious Diseases, The Fifth Medical Center of Chinese PLA General Hospital, Beijing, China

**Keywords:** HBV, T-cell responses, Core, Envelope, Metabolic interventions, Mitochondria-targeted antioxidants, Polyphenolic compounds, ACAT inhibitors, Eosinophil, RDW-CV

## Abstract

**Background:**

Restoration of HBV-specific T cell immunity is a promising approach for the functional cure of chronic Hepatitis B (CHB), necessitating the development of valid assays to boost and monitor HBV-specific T cell responses in patients with CHB.

**Methods:**

We analyzed hepatitis B virus (HBV) core- and envelope (env)-specific T cell responses using in vitro expanded peripheral blood mononuclear cells (PBMCs) from patients with CHB exhibiting different immunological phases, including immune tolerance (IT), immune activation (IA), inactive carrier (IC), and HBeAg-negative hepatitis (ENEG). Additionally, we evaluated the effects of metabolic interventions, including mitochondria-targeted antioxidants (MTA), polyphenolic compounds, and ACAT inhibitors (iACAT), on HBV-specific T-cell functionality.

**Results:**

We found that HBV core- and env-specific T cell responses were finely coordinated and more profound in IC and ENEG than in the IT and IA stages. HBV env-specific T cells were more dysfunctional but prone to respond to metabolic interventions using MTA, iACAT, and polyphenolic compounds than HBV core-specific T-cells. The responsiveness of HBV env-specific T cells to metabolic interventions can be predicted by the eosinophil (EO) count and the coefficient of variation of red blood cell distribution width (RDW-CV).

**Conclusion:**

These findings may provide valuable information for metabolically invigorating HBV-specific T-cells to treat CHB.

**Graphical abstract:**

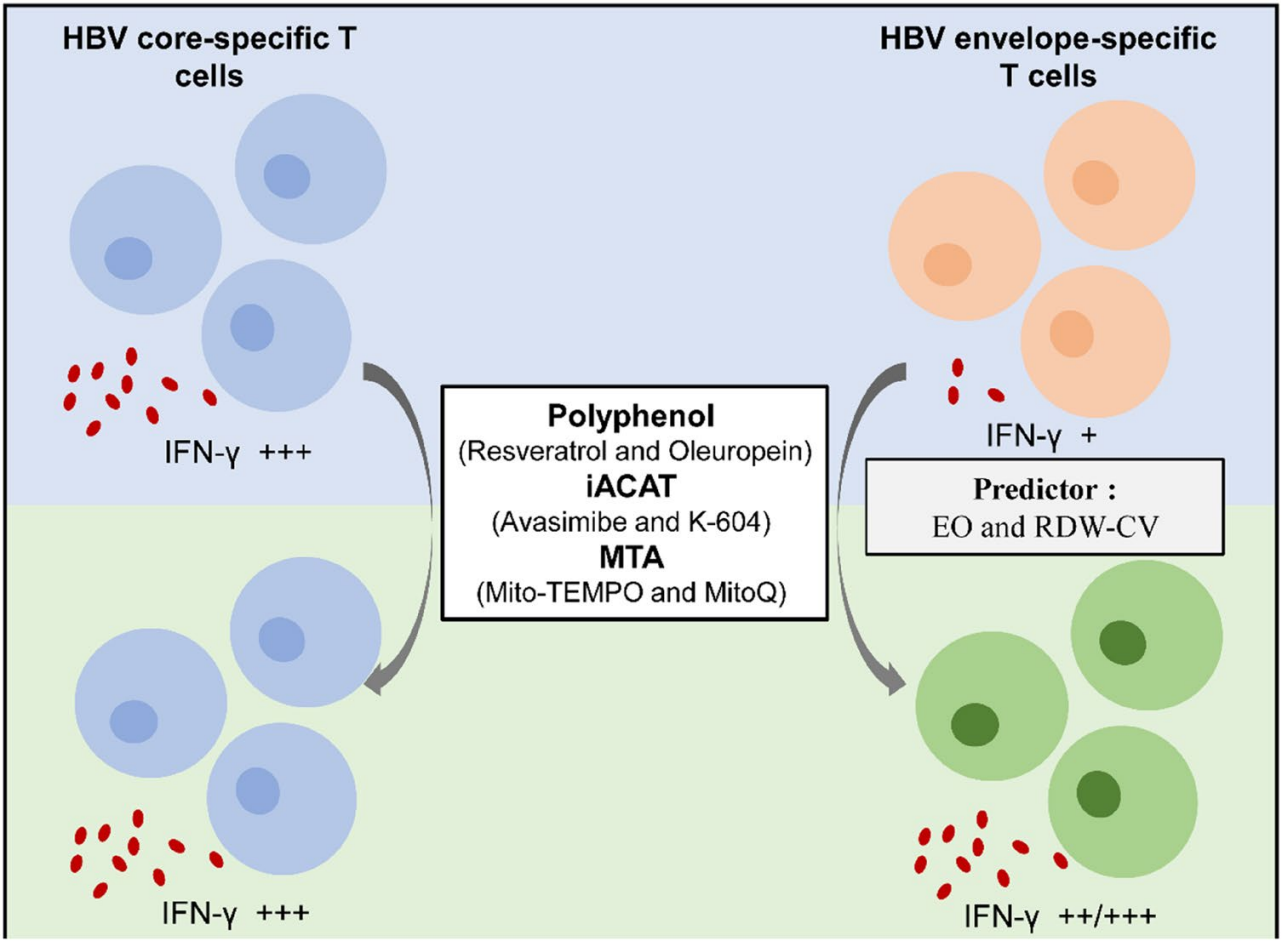

**Supplementary Information:**

The online version contains supplementary material available at 10.1007/s12072-023-10490-4.

## Introduction

Hepatitis B virus (HBV) chronically infects more than 250 million people worldwide and causes an estimated 880,000 deaths a year from liver disease and hepatocellular carcinoma (HCC) [1]. Current antiviral therapies (pegylated interferons (peg-IFN) and nucleos(t)ide analogues (NAs)) provide long-term benefits by suppressing HBV viremia and reducing hepatic necroinflammation [2–4]. However, they cannot eradicate HBV from the liver, have a limited impact on HBsAg levels, and do not eliminate the risk of HCC [2–6]. A promising strategy to achieve a functional cure for chronic HBV infection is to combine potent antiviral agents and immunomodulatory approaches [7, 8].

In adult patients, a vigorous and functionally efficient HBV-specific T-cell response has been observed to be critical for viral clearance during self-resolved acute HBV infection [9–11]. However, in most cases of vertical transmission and infection in early childhood, HBV establishes a chronic infection with a weak, narrow, and delayed T-cell response that is insufficient to eliminate the virus [11, 12]. Additionally, treatment with peg-IFN or long-term NAs resulted in partial recovery of HBV-specific T-cell function [13–15]. Moreover, functional HBV-specific T-cells are needed to control the resurgence of viral replication after the successful cessation of long-term antiviral therapy [16]. Therefore, rebuilding an efficient HBV-specific T-cell response is a rational strategy for treating chronic hepatitis B (CHB) [17].

T-cell-directed therapy development for CHB requires a better understanding of the phenotype and functionality of HBV-specific T-cells. However, such studies are limited by the low frequencies and high exhaustion status of HBV-specific T-cells in the peripheral blood mononuclear cell (PBMC) compartment. Therefore, monitoring and analysis of HBV-specific T-cells ex vivo are difficult for CHB, which usually depends on in vitro expansion [18–21] or enrichment [22–24]. Much effort has been devoted to developing more sensitive assays to measure HBV-specific T-cells ex vivo. For example, programmed cell death ligand 1 (PD-L1) blockade can enhance HBV-specific CD8 + T-cell responses in patients with modest immune exhaustion [25–29]. Targeting T-cell metabolism is a promising strategy because of the increasing evidence linking metabolic pathway dysregulation to T-cell exhaustion [30, 31]. In line with this possibility, restoring mitochondrial function by treatment with antioxidants or polyphenolic compounds elicits HBV-specific T-cell function [32, 33]. Treatment with the acyl-CoA: cholesterol acyltransferase inhibitor (iACAT) improves the responsiveness of HBV-specific CD8 + T-cells to PD-1 blockade [34]. The impact of these metabolic interventions on the quality of HBV-specific T-cell responses in patients with different disease statues of CHB has not been investigated (Table 1).

**Table 1:** Clinical data of patients

Group	Immune tolerance (IT)	Immune activation (IA)	Inactive carrier (IC)	HBeAg-negative hepatitis (ENEG)
Cases	8	9	10	9
Age (years)	34 (26–56)	27 (23–54)	45 (26–60)	49 (30–64)
Sex (M/F)	5/3	5/4	5/5	4/5
HBcrAg (lg U/mL)	8.57 (8.43–8.61)	8.62 (6.19–9.01)	2.00 (2.00–4.83)	5.03 (2.48–7.06)
HBsAg (lg IU/mL)	4.86(4.64–5.04)	4.64 (4.13–5.10)	3.15 (0.16–4.25)	3.31 (1.79–4.28)
HBV DNA (lg IU/mL)	8.32 (8.00–8.63)	8.18(5.73–8.84)	2.38 (1.30–4.95)	4.67 (2.53–7.53)
ALT (U/L)	24.5 (15–37)	116 (42–607)	21.5 (6–36)	94 (43–938)
AST (U/L)	22 (19–29)	90 (31–249)	20.5 (16–28)	47 (36–287)

Continuous variables are presented as median (range)

In this study, we investigated HBV core- and envelope (env)-specific T-cell responses using in vitro expanded PBMCs from patients with CHB exhibiting different disease phases, including immune tolerance (IT), immune activation (IA), inactive carrier (IC), and hepatitis B e antigen (HBeAg)-negative hepatitis (ENEG). Additionally, we evaluated the effects of metabolic interventions, including mitochondria-targeted antioxidants (MTA), polyphenolic compounds, and ACAT inhibitors (iACAT), on HBV-specific T-cell functionality. We also evaluated the potential clinical and virological parameters that can predict the responses of HBV-specific T-cells to metabolic interventions. This study identified metabolic interventions as promising approaches for optimizing HBV-specific T-cell detection and T-cell-based therapies.

## Methods

### Patients

A total of 36 patients with CHB were enrolled at The Fifth Medical Center of Chinese PLA General Hospital, Beijing, China (Table 1). Eight patients displayed clinical, biochemical, and virological evidence of HBeAg + infection (HBsAg positive, HBeAg positive, anti-HBe negative, and anti-HBc positive), with normal alanine transaminase (ALT) levels (≤ 40 U/L), also called immune tolerance (IT) phase. Nine patients exhibited HBeAg + hepatitis infection (HBsAg positive, HBeAg positive, anti-HBe negative, and anti-HBc positive), with abnormal ALT levels (> 40 U/L), also called immune active (IA) or clearance phase. Ten patients exhibited HBeAg- infection (HBsAg positive, HBeAg negative, anti-HBe positive, and anti-HBc positive), with normal ALT levels (≤ 40 U/L), also called inactive carrier (IC) phase. Nine patients exhibited HBeAg- hepatitis (HBsAg positive, HBeAg negative, anti-HBe positive, and anti-HBc positive), with abnormal ALT levels (> 40 U/L), also called HBeAg-negative hepatitis (ENEG) phase. The classification of patients was based on the nomenclature defined by the European Association for the Study of the Liver (EASL) 2017 [35]. All patients were HCV, HDV, and HIV negative, without cirrhosis and hepatocellular carcinoma (HCC). Informed consent was obtained from all the participants.

### Laboratory test

HBV DNA, ALT, aspartate aminotransferase (AST), and other clinical parameters were measured at The Fifth Medical Center of Chinese PLA General Hospital. An iFlash HBsAg assay (YHLO, China) was used for HBsAg quantification. The assay had a calibration range of 0 to 250 IU/mL and a cutoff value of 0.05 IU/mL. The samples were retested at a dilution of 1:500 when qHBsAg levels were more than 250 IU/mL. If HBsAg levels were less than 0.05 IU/mL with a repeat test, the sample was considered non-reactive. Serum HBcrAg levels were quantified using a Lumipulse G1200 automated analyzer (Fujirebio, Japan). Samples were tested according to the manufacturer’s instructions.

### PBMC isolation

PBMCs were isolated from fresh heparinized blood by density gradient centrifugation using Ficoll-Hypaque according to the manufacturer’s instructions. Cells were cryopreserved in liquid nitrogen before further use.

### T cell expansion and treatments

PMBCs were thawed and 1 × 10^6^ cells/mL were cultured at 37 °C in a 5% CO_2_ incubator for 10 d in AIM V (Gibco, USA) supplemented with 5% human serum (Thermo Fisher Scientific, USA) in round-bottomed 96-well plates. Cells were stimulated with 1 μg/mL overlapping peptides of HBV core (PM-HBV-CPULTRA) or HBV envelope (env) (PM-HBV-LEPULTRA) from JPT in the presence or absence of the different compounds tested. These compounds included polyphenols, resveratrol 5 μM and oleuropein 0.25 μM (APExBIO, USA); iACAT, Avasimibe 0.25 μM (Selleck, USA) and K-604 0.05 μM (MCE, USA); and MTA, Mito-TEMPO 5 μM (Merk, Germany) and MitoQ 0.05 μM (MCE). PBMCs were cultured for 10 d in the presence of recombinant IL-2 (20 IU/mL), IL-7 (10 ng/mL), and IL-15 (10 ng/mL) (Biolegend, USA).

### ELISpot

HBV-specific T-cell responses were analyzed using IFN-γ ELISpot assays (Mabtech, Sweden) after 10 d of core- or env-specific polyclonal T-cell expansion. ELISpot plates were washed four times with sterile phosphate-buffered saline (PBS) and blocked with AIM V supplemented with 5% human serum for 30 min at 18–25 ℃. PBMCs were re-stimulated with or without HBV antigen peptides (1 μg/mL) and seeded at a final concentration of 5 × 10^4^ cells/mL per well. The plates were then incubated at 37 °C for 18–24 h. After five washes with sterile PBS, 100 μL biotinylated anti-human IFN-γ (7-B6-ALP) was added to each well. The plates were incubated for 2 h at room temperature. After five washes, 100 μL ready-to-use substrate solution (BCIP/NBT-plus) was added in the dark. After the spots emerged, color development was stopped by washing extensively with distilled water. The plates were air-dried and spots were inspected and counted using an automated ELISpot reader (ChampSpot III, China). The unstimulated control value was subtracted from the peptide-stimulated sample to determine the specific IFN-γ-secreting cells in all the summary data. In this study, wells were considered positive if they had values at least two times above the control wells without stimulation and the number of spot-forming units (SFU) was more than five.

### Intracellular cytokine staining (ICS) and activation-induced marker (AIM) assays

PBMCs were incubated after 10 d of expansion with AIM V alone (control) or core/env peptides (1 μg/mL) for 1 h. Monensin was added to cells for the ICS assays, and not to cells for the AIM assays. Then, PBMCs were incubated for an additional 5 h. For ICS assays, the cells were stained with anti-CD3 BV510 (OKT3) and anti-CD8 FITC (HIT8a) for 30 min at 4 °C. Cells were washed, fixed, permeabilized using the Cytofix/Cytoperm kit, and stained with anti-IFN-γ AF647 (4S.B3), anti-TNF-α BV421 (MAb11), and anti-IL-2 PE-Cy7 (MQ1-17H12) for 40 min at 4 °C. Then cells were washed and analyzed by flow cytometry. For AIM assays, cells were stained with anti-CD3 BV510 (OKT3), anti-CD8 FITC (HIT8a), anti-CD69 BV421 (FN50), CD137 PE-Cy7 (4B4-1), and CD154 PE (24–31) for 30 min at 4 °C. Cells were washed and analyzed by flow cytometry. Samples were analyzed on a FACS Canto II flow cytometer (BD Biosciences, USA) and analyzed using FlowJo software (BD Biosciences).

### Receiver operating characteristic (ROC) curve analysis

Metabolic interventions were considered effective when they increased the env-specific T-cell response levels by two-fold or more and were then divided into two groups, effective or ineffective. Receiver operating characteristic (ROC) curve analysis was performed on the corresponding eosinophil (EO) count and variation of red blood cell distribution width (RCW-CV) of the two groups.

### Statistical analysis

Comparisons between the two groups were performed using the Wilcoxon matched-pairs signed rank (variables from the same person) or Mann–Whitney (variables not from the same person) tests. Correlations between the variables were determined using Spearman’s correlation analysis. Differences were considered statistically significant at *p* < 0.05.

## Results

### HBV-specific T-cell responses in chronic hepatitis B infection

**Fig. 1:**
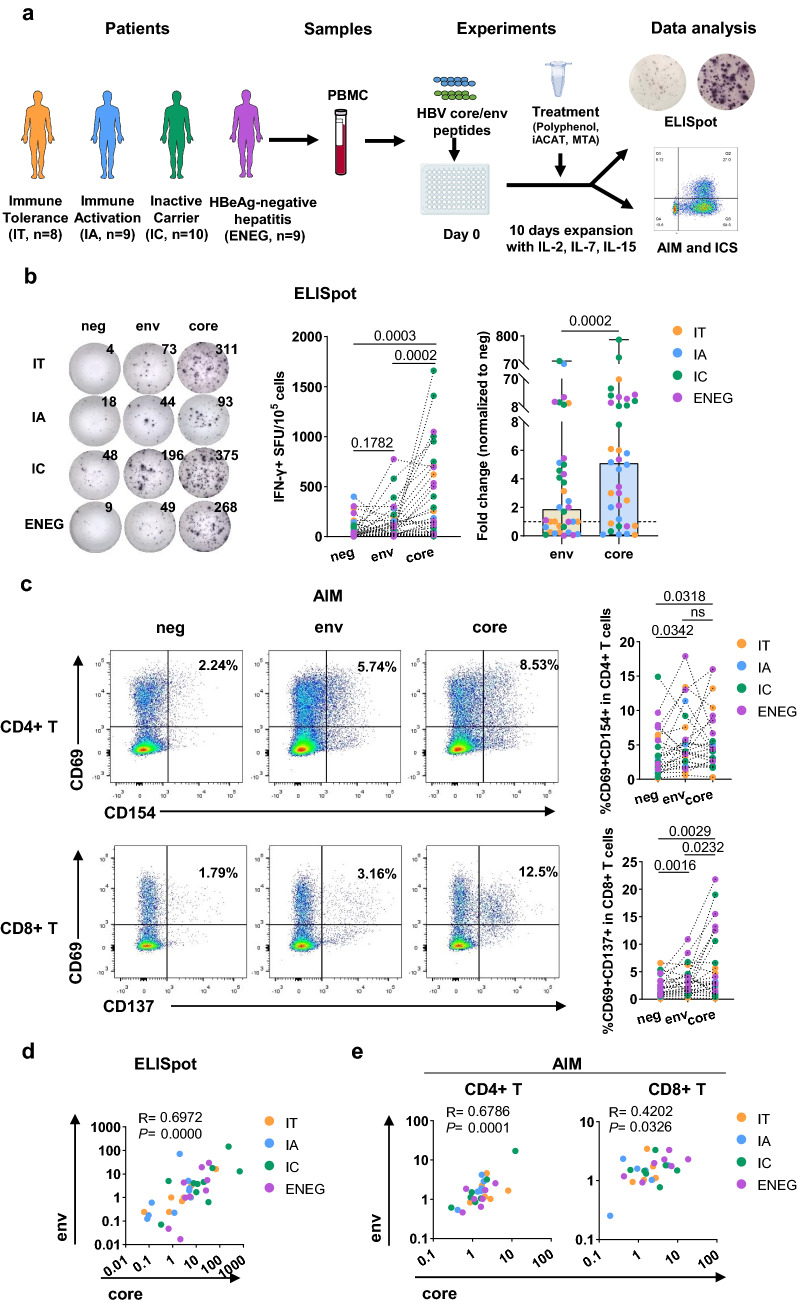
Diagram of the experimental design and the HBV-specific T cell response profiling. **a** Patient enrollment and overview of the study design. **b** IFN-γ ELISpot for core-specific and env-specific T cell responses in patients with CHB. The left showed the representative ELISpot image of IFN-γ + SFU taken from HBV peptides or equal volume DMSO (negative control) restimulation after 10 d of expansion. The middle showed the number of IFN-γ + SFU derived from HBV peptides or DMSO restimulation. The right showed the HBV peptide-induced fold change of IFN-γ production in T cells. **c** AIM expression by core-specific and env-specific CD4 + and CD8 + T cells after 10 d of expansion. **d** The correlation between the levels of responsive T cells to HBV core- and env-peptide stimulation by ELISpot. **e** The correlation between the levels of responsive T cells to HBV core- and env-peptide stimulation by AIM.* P* values are from Wilcoxon matched-pairs signed rank test. Spearman's rank correlation test was used in **d** and **e**. The “ns” represents *P* > 0.05. CHB, chronic hepatitis B; DMSO, Dimethyl sulfoxide; SFU, spot-forming unit; AIM, activation inducer molecule

Recent studies have highlighted that the phenotype and function of HBV-specific T-cells are affected by the targeted epitope and stage of infection [21–23]. Using an adapted peptide-based expansion assay [18–21] (Fig. 1a), HBV-specific T-cell responses were compared to the HBV core and env by conventional antigen-specific T-cell assays (ELISpot, AIM, and ICS) in patients with CHB and different disease statuses (IT, *n* = 8; IA, *n* = 9; IC, *n* = 10; ENEG, *n* = 9). Core-specific and env-specific T-cell responses were detectable upon expansion with core and env peptide pools, respectively, which was consistently observed when T-cell activation was evaluated by ELISpot (Fig. 1b), flow cytometry-based AIM (Fig. 1c), and multiple ICS assays (Fig. S1). Moreover, ELISpot data showed that a significantly higher number of IFN-γ + SFU derived from core peptide pools were detected than that from env (*p* = 0.0002, Fig. 1b), suggesting that env-specific T-cells are more dysfunctional than core-specific T-cells, which is in agreement with previous reports [22, 23, 36]. In the AIM assay, the differences between env- and core-specific T-cells were significant in CD8 + T-cells but not in CD4 + T-cells. These results reveal the heterogeneity of HBV-specific T-cell responses targeting different proteins, with env-specific T-cells being more dysfunctional than core-specific T-cells. The association between core- and env-specific T-cell responses was further assessed. The extent of IFN-γ production by core-specific T-cells correlated positively with that by env-specific T-cells (*r* = 0.6972, *p* < 0.0001, Fig. 1d). Similarly, AIM expression in core- and env-specific T-cells was also positively correlated (CD4 + T: *r* = 0.6786, *p* = 0.0001; CD8 + T: *r* = 0.4202, *p* = 0.0326; Fig. 1e). ICS detection showed that the IL-2 secretion capacity of core-specific CD8 + T-cells was positively correlated with that of env-specific CD8 + T-cells (*r* = 0.4748, *p* = 0.0191, Fig. S2).

**Fig. 2:**
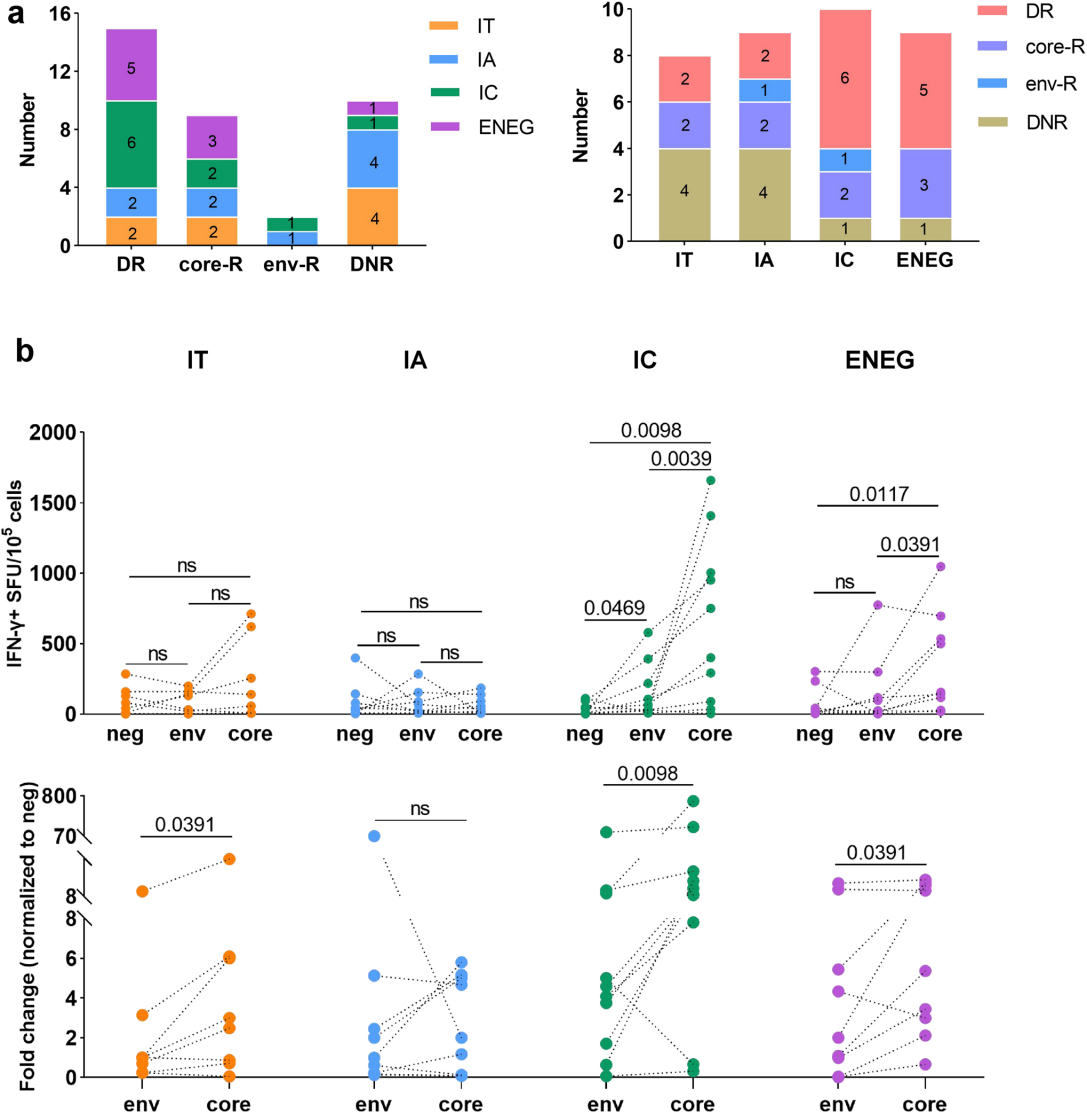
Characters of HBV-specific T cell responses in patients with different disease stages detected by ELISpot. **a** The number and distribution of DNR, core-R, env-R, and DR in patients with CHB at different disease stages. **b** The upper panel showed the IFN-γ secretion by core- and env-specific T cells from patients with CHB at different disease stages. The bottom panel showed the HBV peptide-induced fold change of IFN-γ production in T cells. Comparison between groups were calculated using Wilcoxon matched-pairs signed rank tests. The “ns” represents *P* > 0.05. DNR, double non-responses; core-R, core only responses; env-R, env only responses; DR, double responses

Next, patients were divided into four groups based on their T-cell reactivity to the core and env: double non-responders (DNR), core-only responders (core-R), env-only responders (env-R), and double responders (DR). The results showed that 10 (27.78%), nine (25%), two (5.56%), and 15 (41.66%) patients had DNR, core-R, env-R, and DR, respectively (Fig. 2a). DNR patients were preferentially allocated to the IT and IA stages, while most patients in the IC and ENEG stages showed detectable core- and env-specific T-cell responses (Fig. 2a). Additionally, ELISpot assay showed that the aforementioned stronger responses of core-specific T-cells than that of env-specific T-cells were mainly observed in the IC and ENEG stages (Fig. 2b). Moreover, increased expression of CD69 + CD137 + on core- and env-specific CD8 + T-cells was observed in the ENEG stage analyzed by AIM assay (Fig. S3). ICS assay showed that the secretion of IFN-γ, TNF-α and IL-2 by core- and env-specific T-cells were the most apparent in ENEG among all stages (Fig. S4). In summary, these results suggest that T-cell responses targeting different HBV proteins are finely coordinated and more profound in patients with HBeAg- than in patients with HBeAg + CHB.

### Impact of viral products on HBV-specific T-cells

**Fig. 3:**
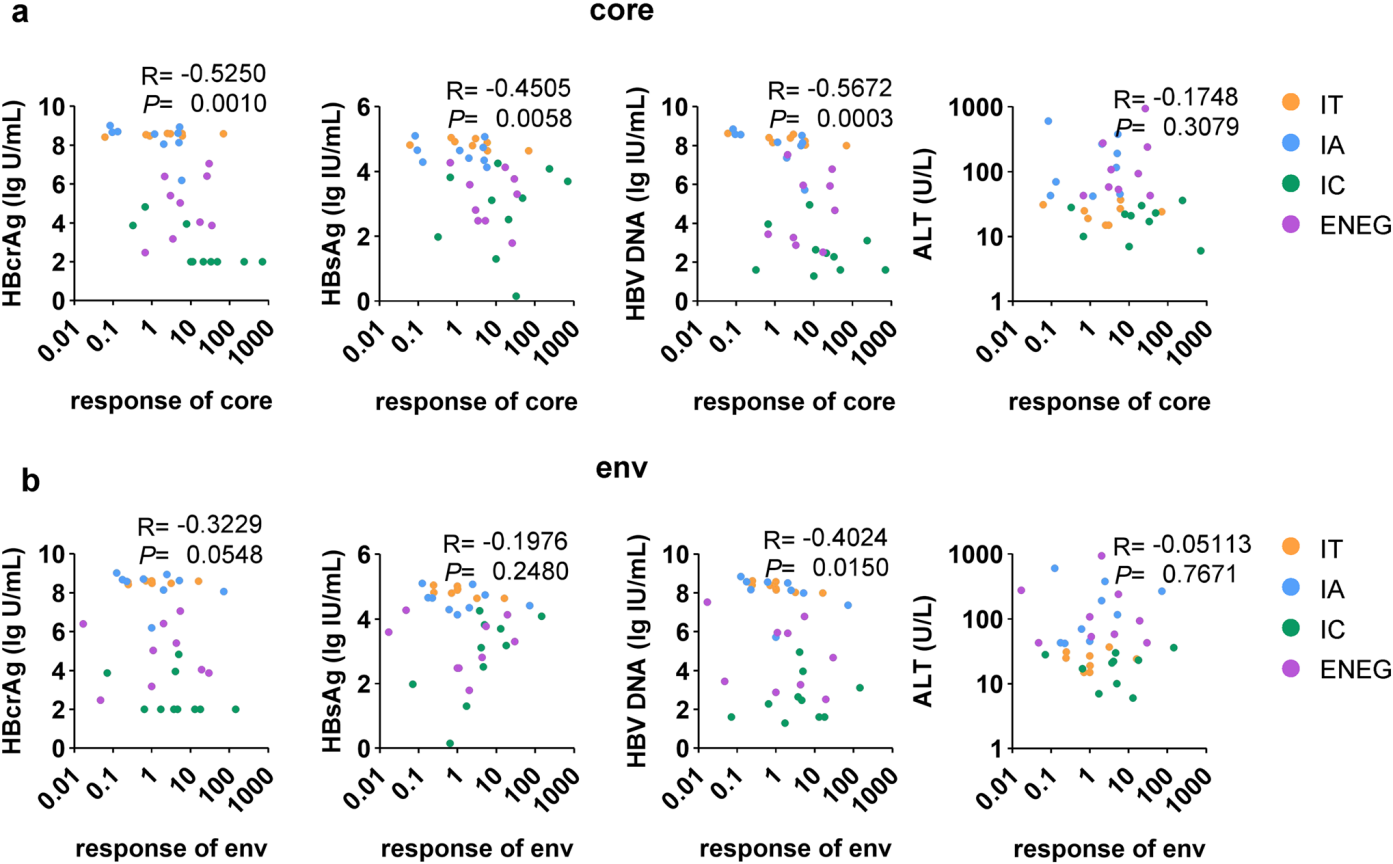
The correlations between HBV-specific T cell responses and clinical parameters. **a** The correlations between core-specific IFN-γ + SFU and serum levels of HBcrAg, HBsAg, HBV DNA, and ALT. **b** The correlations between env-specific IFN-γ + SFU and serum levels of HBcrAg, HBsAg, HBV DNA, and ALT. Spearman's rank correlation analysis

The association between HBV-specific T-cell function and virological parameters is important for understanding the interplay between the host immune system and the virus [37]. The relationship between the responses of antigen-specific T-cells and virological and clinical parameters including HBcrAg, HBsAg, HBV DNA, and ALT was analyzed. The function of core-specific T-cells was confirmed to be negatively correlated with serum levels of HBcrAg, HBsAg, and HBV DNA [19, 21, 38, 39] (Fig. 3a). Furthermore, the responses of env-specific T-cells tended to be negatively correlated with HBV DNA and HBcrAg, but there was no significant correlation between env-specific T-cell responses and HBsAg levels (Fig. 3b). This may be due to severe functional defects in env-specific T-cells in patients with CHB.

Interestingly, when the patients were segregated into different disease stages, the negative correlation between HBV-specific T-cell responses and HBV DNA levels were mainly observed in the IT and IA stages (Fig. S5-6). Moreover, the core-specific T-cell responses were negatively correlated with HBcrAg levels, while the env-specific T-cell responses were positively correlated with HBsAg levels in the IC stage (Fig. S5-6). The different impacts of core and env on their cognate T-cells might be explained by heterogeneities regarding the quantity or cell source of these antigens[40].

### Impact of metabolic interventions on the responses of HBV-specific T-cells

**Fig. 4:**
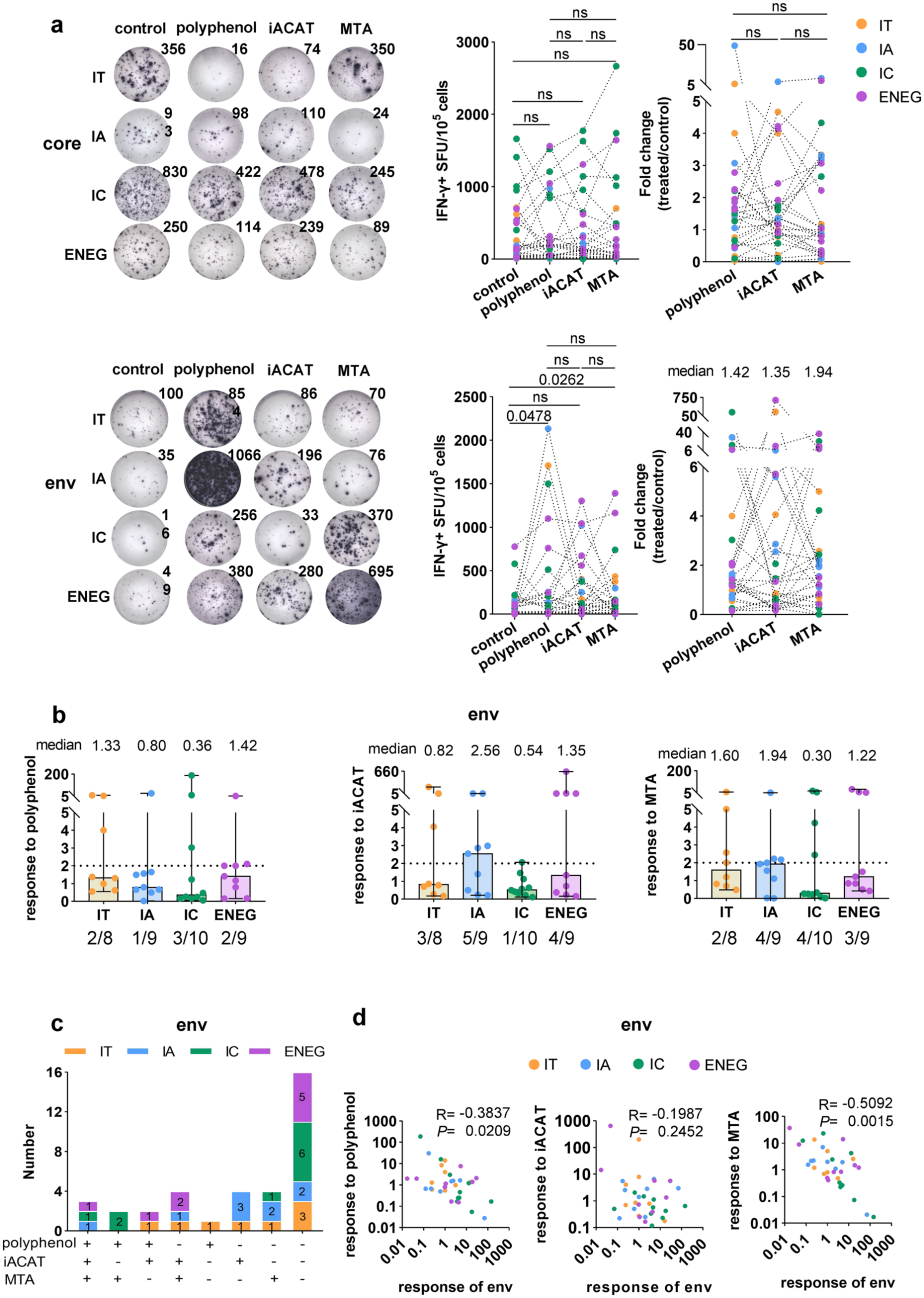
Effect of treatments on HBV-specific T cells. PBMCs from patients with CHB were expanded for 10 d ex vivo with HBV core or env peptides in the presence or absence of three different treatment types. **a** IFN-γ ELISpot for core- and env-specific T cell responses to polyphenol, iACAT, and MTA. On the left were representative ELISpot images of IFN-γ + SFU produced by HBV-specific T cells with or without treatments. The middle was the number of IFN-γ + SFU derived from HBV-specific T cells with or without treatments. The right showed the treatment-induced fold change of IFN-γ levels in T cells. **b** The levels of env-specific T cells response to treatments in different disease stages. The medians are at the upper of the graph. The fractions at the bottom of the graph show the effective intervention rate. **c** The number of responders to metabolic interventions. The “ + ” indicates that the patient's env-specific T cells are responding to a metabolic intervention; the “-” indicates that the patient's env-specific T cells are not responding to a metabolic intervention. **d** The correlation between the levels and degrees of env-specific T cell responses to treatments using Spearman's rank correlation analysis. Differences between treatments in **a** and **b** were evaluated using Wilcoxon matched-pairs signed rank and unpaired nonparametric Mann–Whitney tests, respectively. The “ns” represents *P* > 0.05. Control: peptides only; polyphenol: peptides + polyphenol; iACAT: peptides + iACAT; MTA: peptides + MTA

There is accumulating evidence linking the dysregulation of specific metabolic pathways to T-cell exhaustion, encouraging the targeting of T-cell metabolism as a potential therapy. Similar to HBV infection, studies have shown that metabolic approaches, such as polyphenols, ACAT inhibitors, and mitochondrion-targeted antioxidants, can improve the response of HBV-specific T-cells [32–34]. The utility and clinical relevance of metabolic interventions in CHB were further evaluated by comparing the effects of these intervention reagents on T-cells with different antigen specificities in patients with CHB. The intervention reagents did not exhibit significant effects on core-specific T-cells, but improved the responses of env-specific T-cells, with average fold changes of 10.13, 31.76, and 5.00 for polyphenol, iACAT, and MTA treatments, respectively (Fig. 4a). However, it seems that the responses of env-specific T-cells to interventions were not associated with CHB stages (Fig. 4b). The env-specific T-cells from IA and ENEG stages tended to be more sensitive to iACAT and MTA treatments than other stages (Fig. 4b).

Furthermore, env-specific T-cells from more than 50% (20 of 36) individuals were able to respond to one or more treatments (Fig. 4c). Moreover, the env-specific T-cell response levels were negatively correlated with their fold induction upon metabolic intervention with polyphenol or MTA (Fig. 4d). Since env-specific T-cells were more dysfunctional than core-specific T-cells, these results suggest that metabolic interventions with polyphenol or MTA might selectively revert deeply exhausted T-cells without affecting their functionally intact counterparts.

### Impact of clinical parameters on the env-specific T-cell response to treatments

**Fig. 5:**
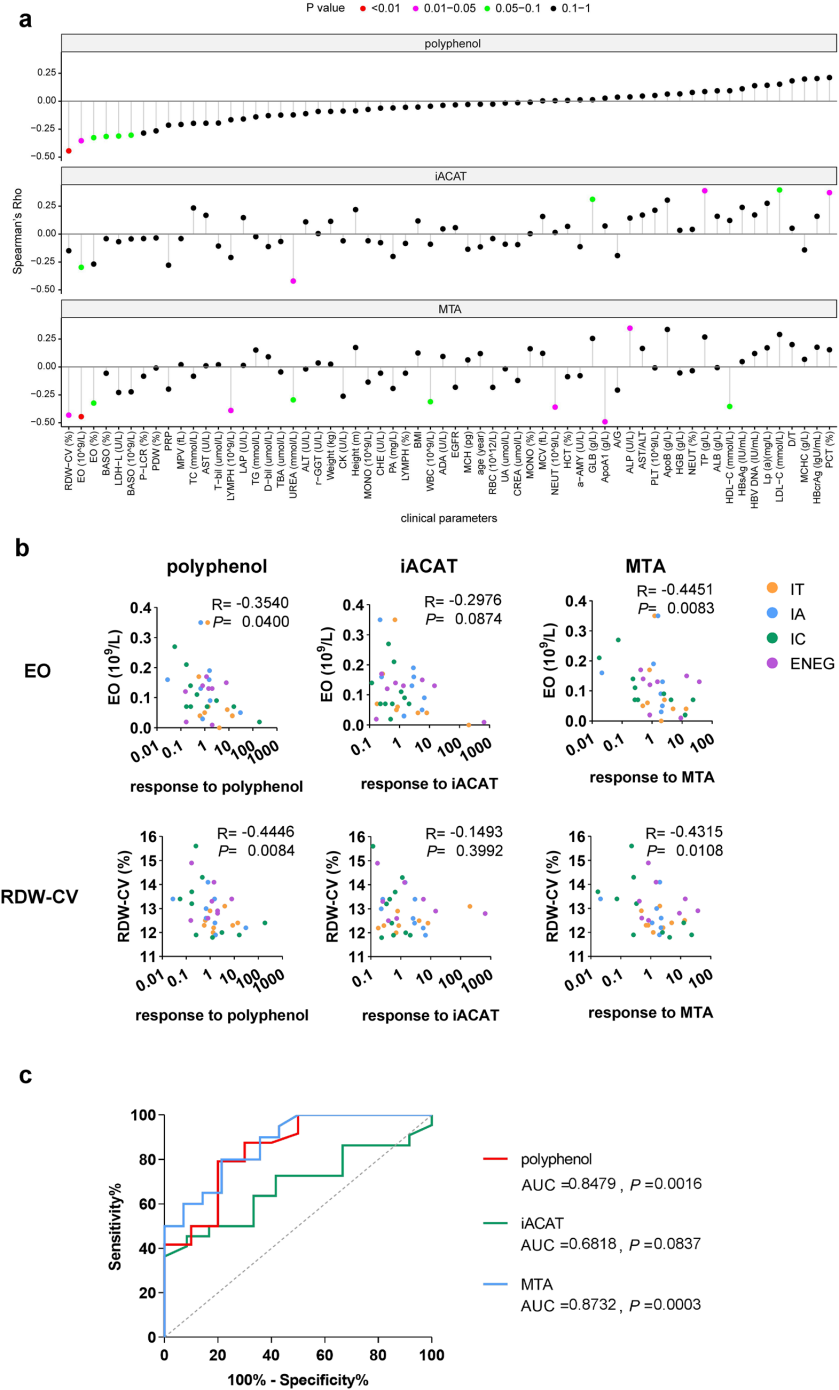
Relationship between clinical parameter levels and env-specific T cell responses to metabolic interventions. **a** Correlation analysis of clinical parameters and the responsiveness of env-specific T cells to treatments. **b** The correlation between the levels of env-specific T cell responses to treatments and the levels of EO and RDW-CV. **c** Receiver operating characteristics curve for predicting responsiveness of env-specific T cells to the combination of EO and RDW-CV. Spearman's rank correlation test was used to compare data in **a** and **b**. EO: eosinophil number; RDW-CV: red blood cell distribution width

The experimental procedures used to monitor specific T-cell responses are complex, time-consuming, and difficult to apply in daily clinical settings. To determine whether there were convenient surrogate markers for the responsiveness of env-specific T-cells to metabolic interventions in patients with CHB, patients were divided into two groups based on their env-specific T-cell reactivity to the metabolic interventions: non-responder and responder. We analyzed the differences in virological and clinical parameters, including HBcrAg, HBsAg, HBV DNA, age, ALT, and AST, between the two groups. Among these, we observed that the ALT and AST levels were slightly lower in polyphenol-responders (Fig S7). Then, we analyzed the relationship between the env-specific T-cell response levels to the treatments and clinical parameters (Fig. 5a and Table S1). We found that both the EO number and RDW-CV were negatively associated with the responsiveness of env-specific T-cells to polyphenols and MTA (Fig. 5b). The predictive effects of these two indicators were further explored by performing ROC curve analysis. The area under the curve (AUC) of polyphenol (AUC = 0.8479, *p* = 0.0016) and MTA (AUC = 0.8732, *p* = 0.0003) was significant (Fig. 5c). Altogether, EO and RDW-CV are associated with the env-specific T-cell response levels to intervention reagents. These could reflect the response preferences of env-specific T-cells to different types of metabolic interventions, which are beneficial for developing personalized therapies.

## Discussion

HBV-specific T-cells play a crucial role in antiviral protection and their dysfunction is associated with HBV persistence. Restoration of HBV-specific T-cell immunity holds promise for HBV cure, but the lack of consistent assays impedes relevant research to measure ex vivo T-cell responses in patients with CHB. Using an adapted peptide-based ELISpot assay, the frequencies of core- and env-responsive T-cells in patients with CHB and their responsiveness to metabolic interventions were analyzed. We found that HBV env-specific T-cells were more dysfunctional, but prone to metabolic interventions by MTA, iACAT, and polyphenolic compounds. Our findings provide valuable data for optimizing HBV-specific T-cell detection and targeting T-cell metabolism for CHB treatment.

Recent studies show that CD8 + T-cells recognizing different HBV epitopes in various proteins exhibit distinct phenotypic and functional features, with env-specific CD8 + T-cells being more dysfunctional than polymerase- or core-specific CD8 + T-cells [22, 23, 41]. In our study, results using broad peptide pools showed a similar pattern to those of the aforementioned studies using HLA-restricted peptide epitopes. Notably, the differences observed between core- and env-specific T-cell responses were mainly attributed to patients who were HBeAg negative (IC and ENEG groups), which might be due to the low response rate for both antigens in patients who were HBeAg positive (IT and IA groups). Moreover, by the simultaneous detection of AIM markers and multiparameter intracellular cytokine staining, we demonstrated that this dichotomy of HBV-specific CD8 + T-cell responses to different HBV proteins is consistent in both CD8 + T and CD4 + T-cells. Env-specific T-cells were more responsive to metabolic interventions than core-specific T-cells, which were more significant for those whose env-responsive T-cells were compromised. This differs from the PD-1/PD-L1 blockade in earlier studies, in which functional restoration was primarily detected on core- and polymerase-specific T-cells, but much less on env-specific T-cells [20, 25, 42]. Notably, env-specific CD8 + T-cells are crucial for CD8 + T-cell-mediated effects on HBV replication and hepatitis [43]. Therefore, metabolic interventions might be superior to or work synergistically with checkpoint inhibitors to restore anti-HBV activity.

The mechanisms underlying the heterogeneity of HBV-specific CD8 + T-cells are not yet well understood. Recent studies have highlighted that the different functional features of CD8 + T-cells targeting distinct HBV proteins might be relevant to the antigenic load and duration of antigen exposure [19, 42]. This study found that HBcrAg and HBV DNA levels were negatively correlated with core-specific and env-specific T-cell responses. In contrast, HBsAg levels were not associated with env-specific T-cell responses. Active HBV replication can contribute to the pool of HBcrAg, HBV DNA, and HBsAg, while HBsAg can be additionally derived from integrated HBV DNA, which constitutes the primary source in IC and ENEG patients. Therefore, our results suggest that active HBV replication promotes T-cell exhaustion. An alternative explanation for the inverse correlation between HBV-specific T-cell responses and HBV viral products is that HBV-specific T-cells might favor virus control and predict the prognosis of HBV infection. These results reinforce the rationale that interventions restoring HBV-specific T-cell responses might reduce viral antigens and a functional cure.

Finite therapy for CHB is an unmet clinical need. Many studies have explored the long-term outcomes after NA treatment cessation among patients who have achieved long-term viral suppression on treatment [44]. Unexpectedly, some patients can achieve HBsAg loss after stopping antiviral therapy, which rarely occurs with sustained NA treatment [35, 36, 45]. Moreover, the characteristics of HBV-specific T-cells could serve as promising predictors of HBV control [39, 46–48]. However, its clinical application is limited by the lack of valid assays able to monitor HBV-specific T-cell responses. Alternatively, we identified EO and RDW-CV, two standard parameters for routine blood tests, as potential indicators for predicting env-specific T-cell responses to metabolic interventions.

There are some limitations in our study. For example, we enrolled treatment-naive CHB patients rather than those with antiviral therapy. The dynamics of HBV-specific T-cells and whether patients who respond to metabolic interventions are more likely to achieve serologic conversion to HBeAg or HBsAg remain unclear. Further longitudinal studies with supplement liver samples[49–51] are needed to understand the metabolic characteristics of HBV-specific T-cells during antiviral treatment. Meanwhile, in-depth research of the metabolic mechanisms of HBV-specific T-cells is also required to develop new drugs to improve HBV-specific T-cell function.

Nevertheless, we found that HBV-specific T-cells have distinct functional capacities and responses to metabolic interventions, which are influenced by the protein targets and stages of infection. These findings may help monitor HBV-specific T-cell responses, highlighting the significance of metabolic interventions for restoring HBV-specific CD8 + T-cells in patients with CHB.

### Supplementary Information

Below is the link to the electronic supplementary material.

Supplementary file1 (PDF 1262 KB)

## Data Availability

All data supporting the findings in this study are available from the corresponding author upon reasonable request.

## Abbreviations

HBV: Hepatitis B virus
CHB: Chronic hepatitis B
PBMCs: Peripheral blood mononuclear cells
IT: Immune tolerance
IA: Immune activation
IC: Inactive carrier
ENEG: HBeAg-negative hepatitis
MTA: Mitochondria-targeted antioxidants
iACAT: Acyl-CoA: cholesterol acyltransferase inhibitor
EO: Eosinophil
RDW-CV: Red blood cell distribution width
IFN-γ: Interferon γ
HCC: Hepatocellular carcinoma
peg-IFN: Pegylated interferons
NAs: Nucleos(t)ide analogues
PD-L1: Programmed cell death ligand 1
PD-1: Programmed cell death protein 1
PLA: People's Liberation Army
EASL: European Association for the Study of the Liver
HCV: Hepatitis C virus
HDV: Hepatitis D virus
HIV: Human immunodeficiency virus
ALT: Alanine transaminase
AST: Aspartate aminotransferase
IL-2: Interleukin-2
IL-7: Interleukin-7
IL-15: Interleukin-15
ELISpot: Enzyme-linked immunosorbent spot
PBS: Phosphate-buffered saline
SFU: Spot-forming units
ICS: Intracellular cytokine staining
AIM: Activation-induced marker
TNF-α: Tumor necrosis factor α
DNR: Double non-responders
core-R: Individuals who respond to core but not env
env-R: Individuals who respond to env but not core
DR: Double responders
ROC curve: Receiver operating characteristic curve
AUC: Area under the curve
HLA: Human leukocyte antigen
DMSO: Dimethyl sulfoxide
