# Supplementary material for: Metabolic interventions improve HBV envelope-specific T-cell responses in patients with chronic hepatitis B
Source: Hepatol Int. 2023 Mar 28;17(5):1125–38. doi: 10.1007/s12072-023-10490-4 (PMC10522531; doi:10.1007/s12072-023-10490-4)
Supplement: Supplementary file 1 — Supplementary file1 (PDF 1262 KB) [file 12072_2023_10490_MOESM1_ESM.pdf]

Metabolic interventions improve HBV envelope-specific T-cell responses in patients with chronic hepatitis B

Authors:

Yu-Long Fu [1, 2, *], Shuang-Nan Zhou [2, *], Wei Hu [2], Jing Li [2], Ming-Ju Zhou [2], Xiao-Yu Li [2], You-Yuan Wang [2], Peng Zhang [2], Si-Yuan Chen [2], Xing Fan [2], Jin-Wen Song [2], Yan-Mei Jiao [2], Ruonan Xu [2], Ji-Yuan Zhang [2], Cheng Zhen [2], Chun-Bao Zhou [2], Jin-Hong Yuan [2], Ming Shi [2], Fu-Sheng Wang [1, 2, #], Chao Zhang [2, #]

Affiliations:

[1]Savaid Medical School, University of Chinese Academy of Sciences, Beijing, China.

[2]Senior Department of Infectious Diseases, The Fifth Medical Center of Chinese PLA General Hospital, Beijing, China.

*These authors shared co-first authorship.

#Correspondence to:

Fu-Sheng Wang, Senior Department of Infectious Diseases, The Fifth Medical Center of Chinese PLA General Hospital, Beijing, China. fswang302@163.com; and Chao Zhang, Senior Department of Infectious Diseases, The Fifth Medical Center of Chinese PLA General Hospital, Beijing, China. zhangch302@163.com.

Fig.S1

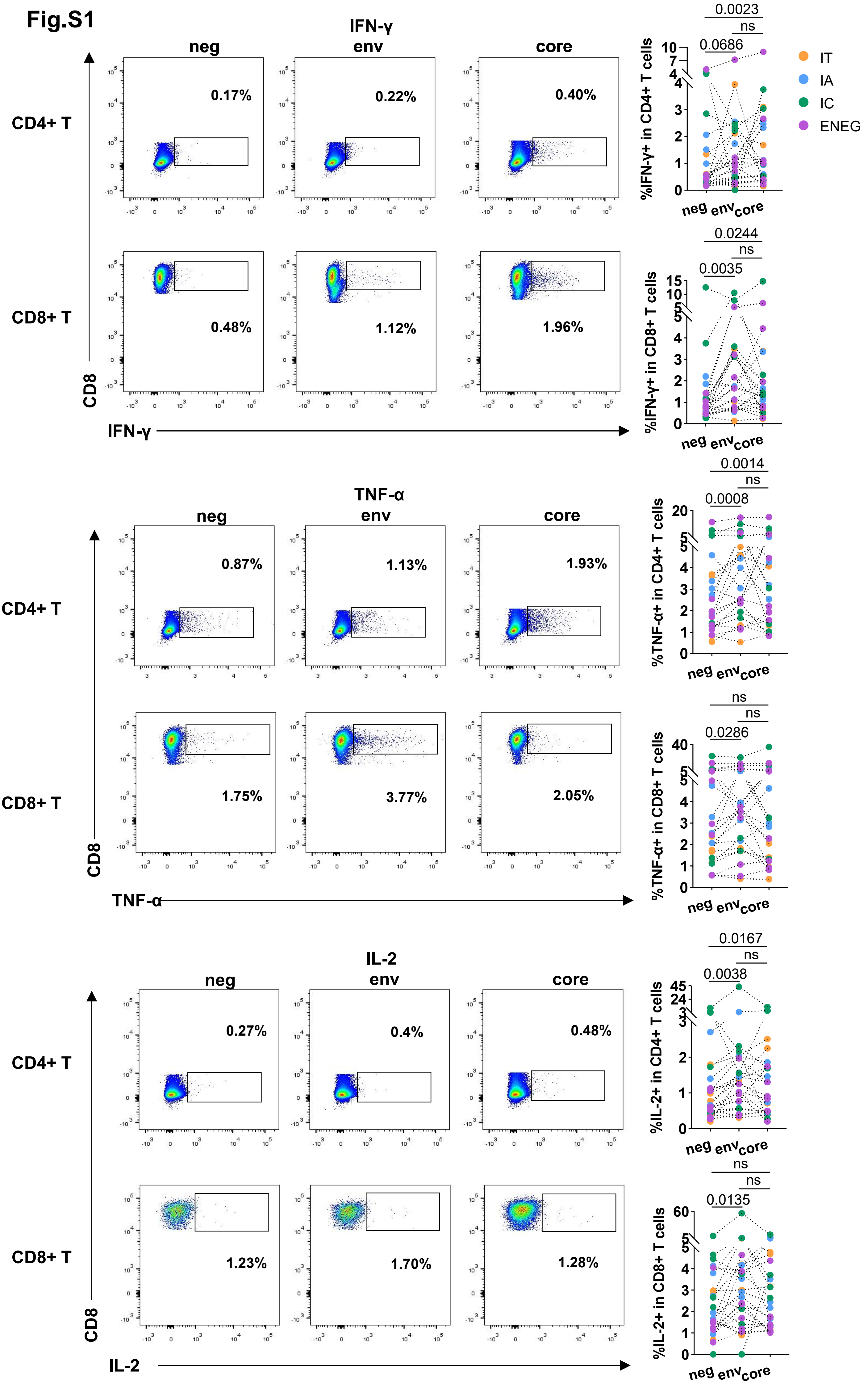
IFN-γ
neg
env
core
CD4+ T
CD8+ T
0.17%
0.22%
0.40%
0.48%
1.12%
1.96%
CD8
IFN-γ
%IFN-γ+ in CD4+ T cells
%IFN-γ+ in CD8+ T cells
0.0023
ns
0.0686
0.0244
0.0035
IT
IA
IC
ENEG
TNF-α
0.87%
1.13%
1.93%
1.75%
3.77%
2.05%
%TNF-α+ in CD4+ T cells
%TNF-α+ in CD8+ T cells
0.0014
0.0008
0.0286
IL-2
0.27%
0.4%
0.48%
1.23%
1.70%
1.28%
%IL-2+ in CD4+ T cells
%IL-2+ in CD8+ T cells
0.0167
0.0038
0.0135

Fig.S1 The percentages of IFN-γ-, TNF-α-, and IL-2-positive core-specific and env-specific CD4+ and CD8+ T cells after 10 days expansion. P values represent Wilcoxon matched-pairs signed rank test.

Fig.S2

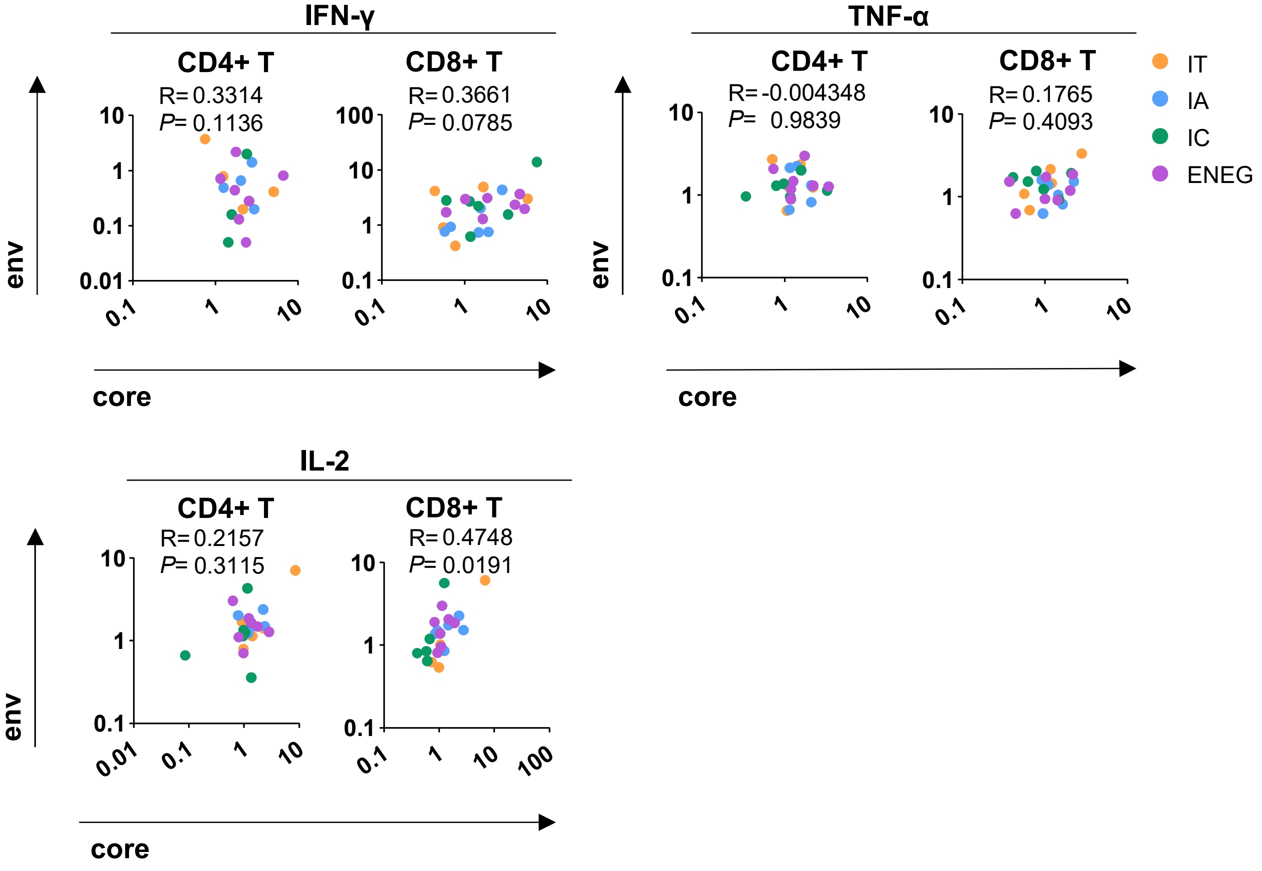

Fig.S2 The correlation between HBV core and env peptide-induced variations (fold change) of IFN-γ, TNF-α, and IL-2 levels in T cells detected by flow cytometry using Spearman's rank correlation analysis.

Fig.S3

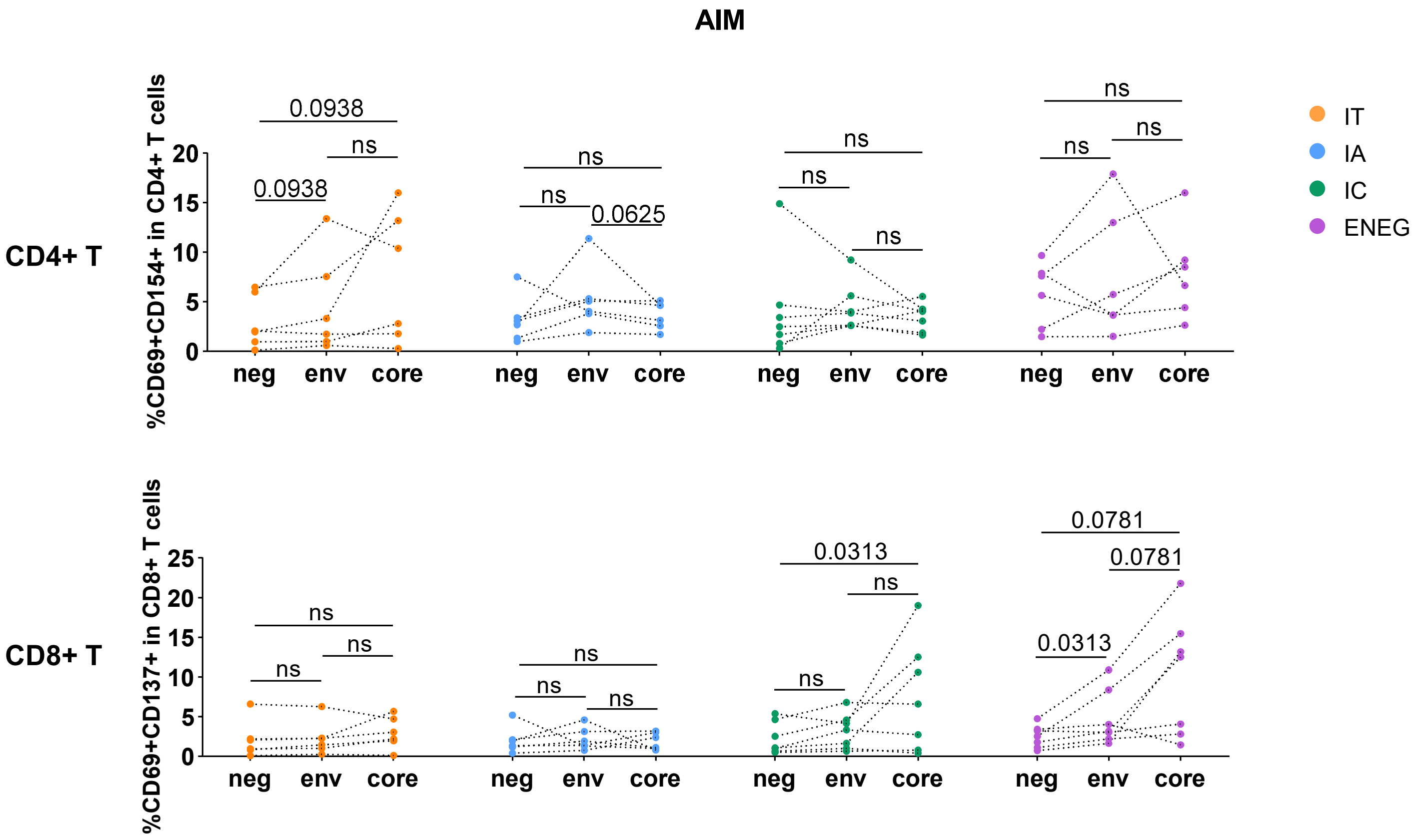

Fig.S3 AIM expression by core- and env-specific CD4+ and CD8+ T cells from patients with CHB at different disease stages. Comparison between groups were calculated using Wilcoxon matched-pairs signed rank tests.

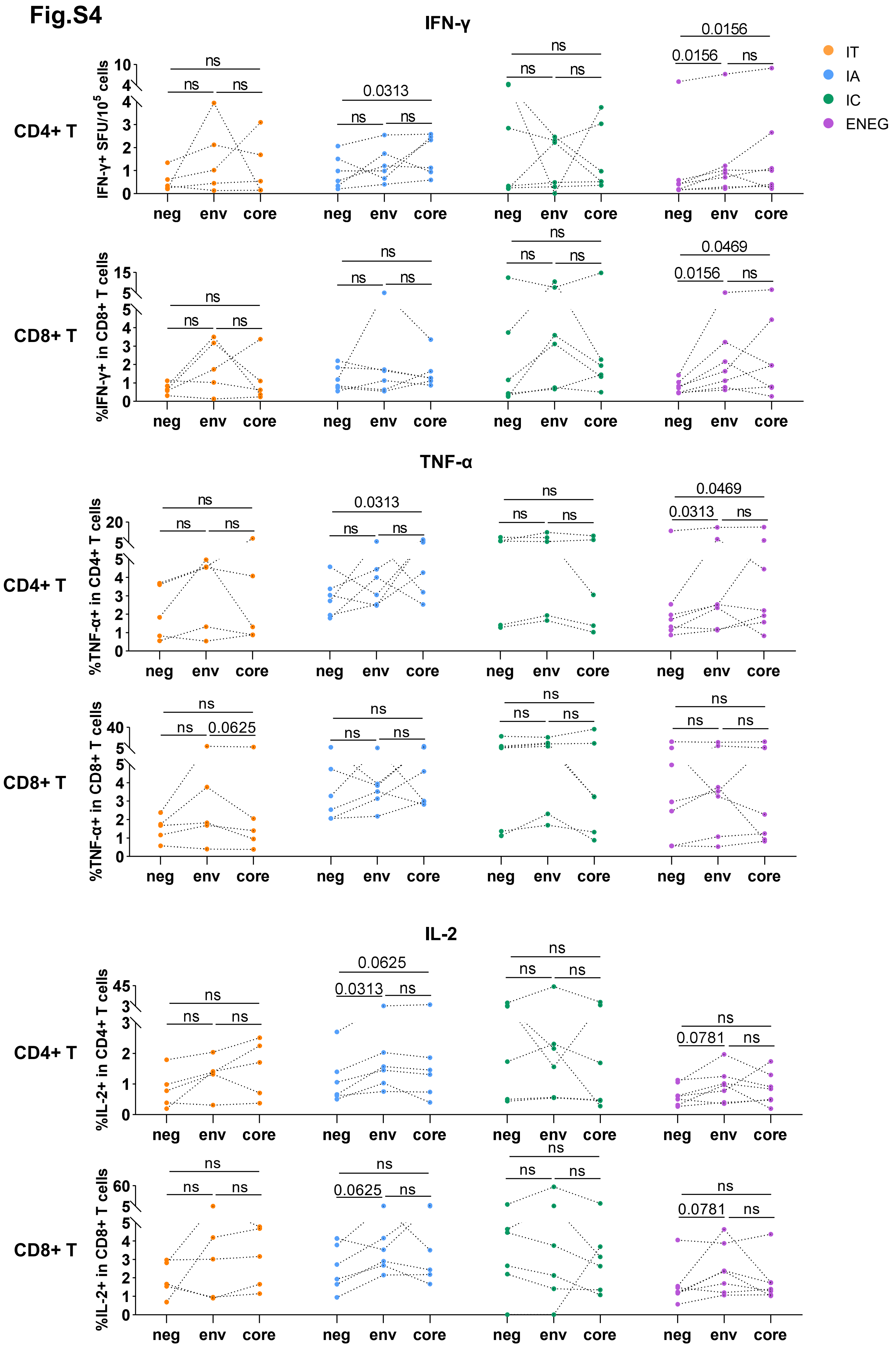

Fig.S4 IFN-γ, TNF-α, and IL-2 secretion by core- and env-specific CD4+ and CD8+ T cells from patients with CHB at different disease stages. Comparison between groups were calculated using Wilcoxon matched-pairs signed rank tests.

Fig.S5

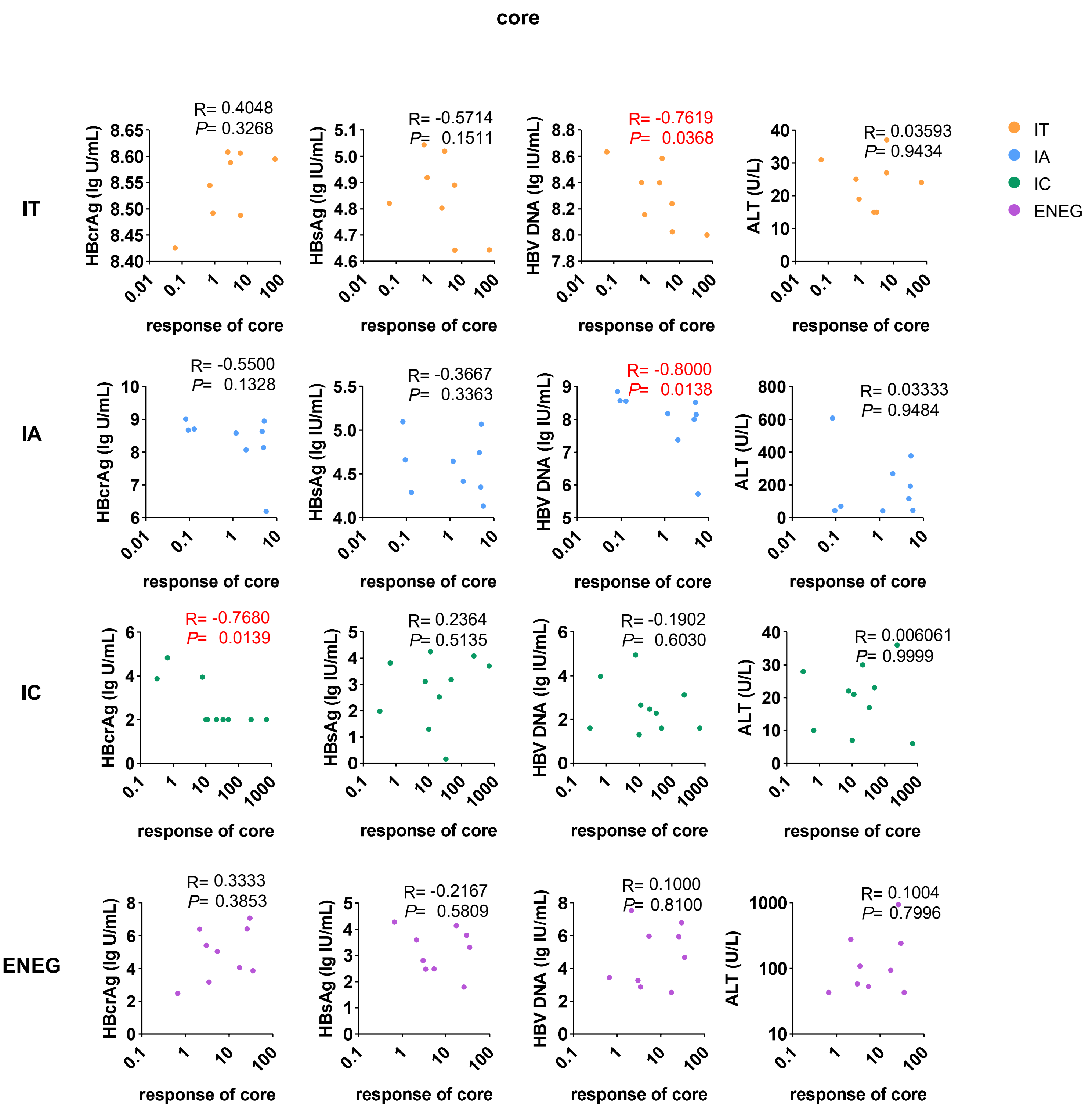

Fig.S5 The correlation between core-specific IFN-γ+ SFU and serum levels of HBcrAg, HBsAg, HBV DNA, and ALT in patients with different disease stages using Spearman's rank correlation analysis.

Fig.S6

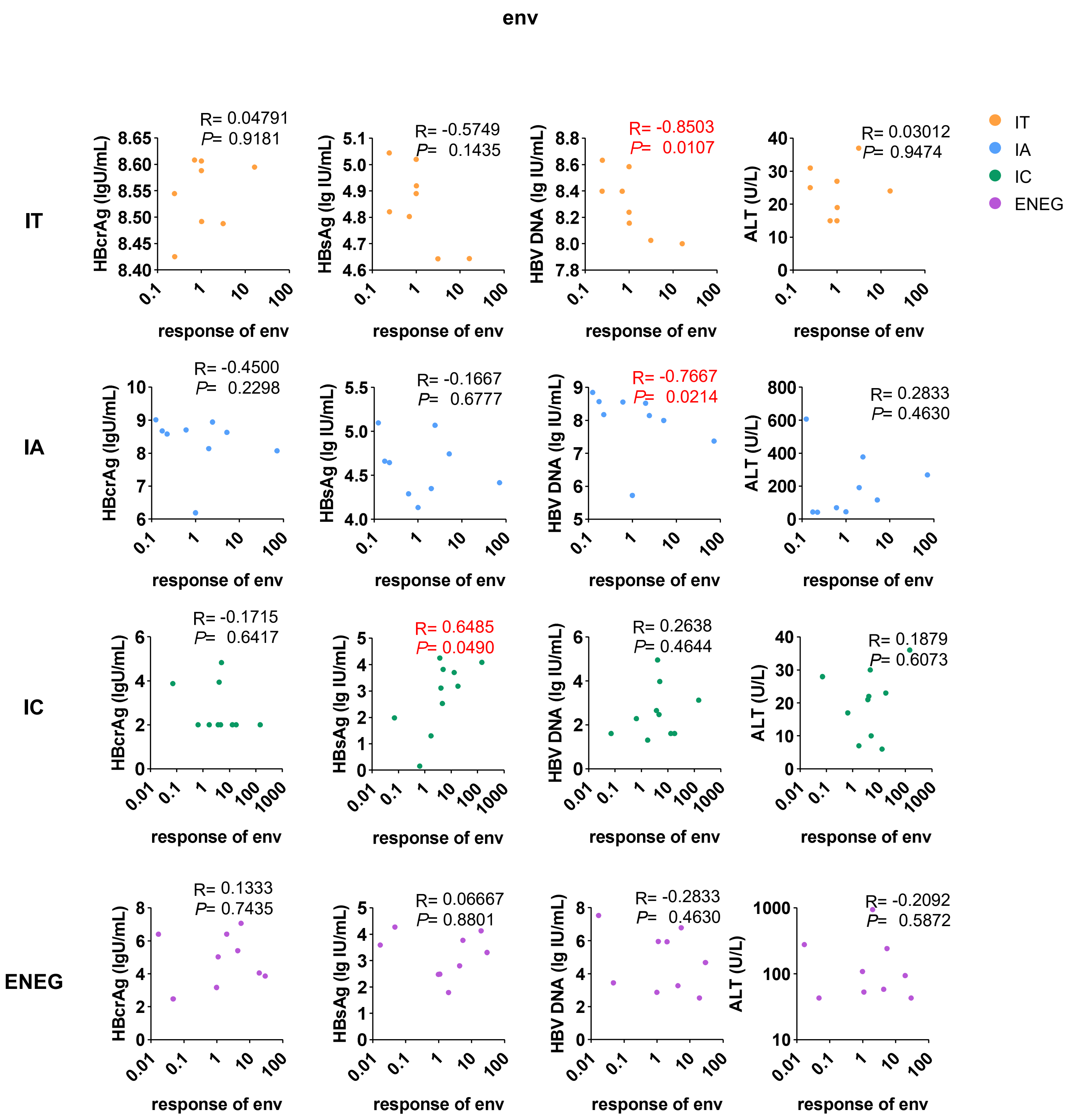

Fig.S6 The correlation between env-specific IFN-γ+ SFU and serum levels of HBcrAg, HBsAg, HBV DNA, and ALT in patients with different disease stages using Spearman's rank correlation analysis.

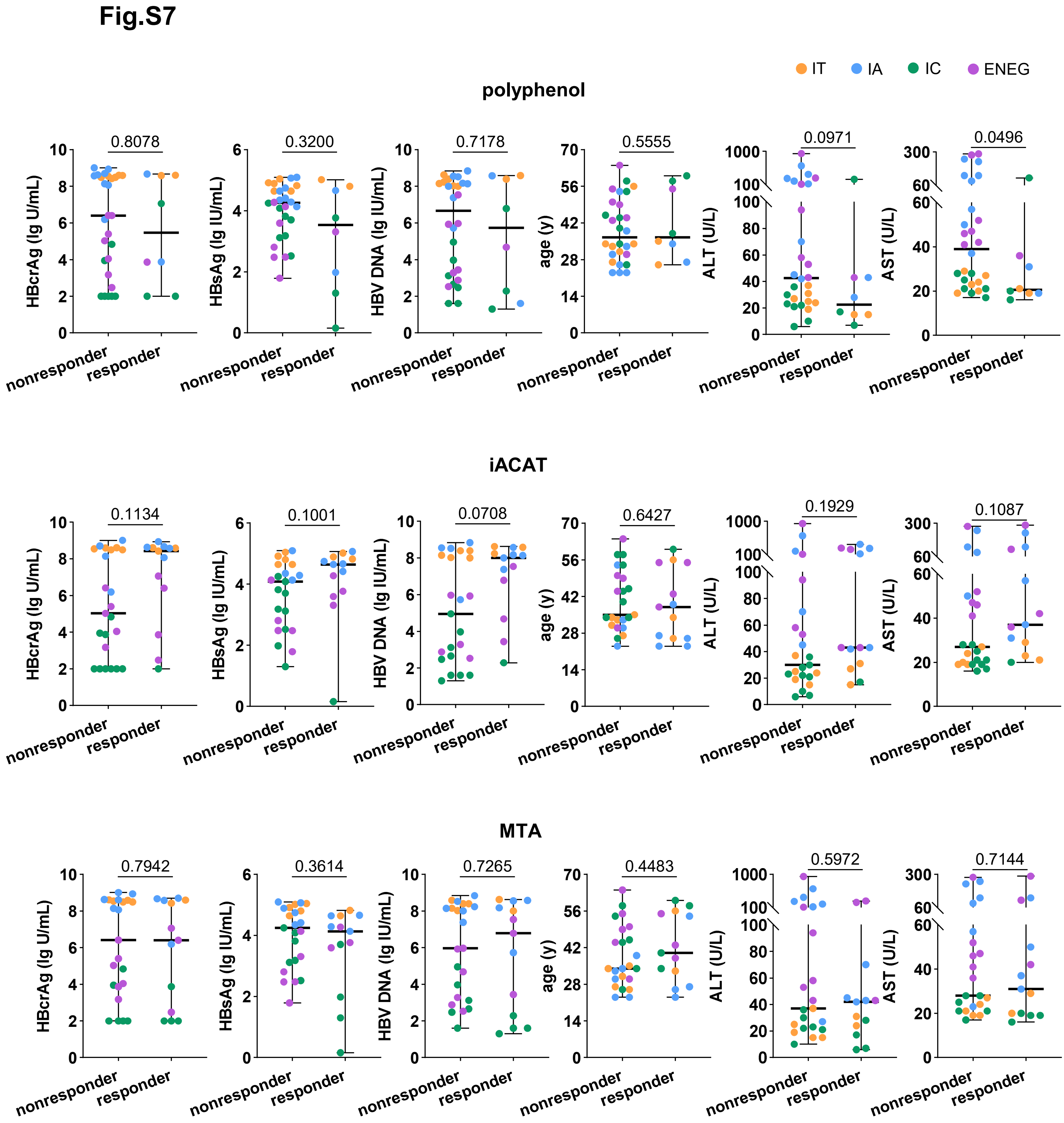

Fig.S7 The levels of HBcrAg, HBsAg, HBV DNA, age, ALT, and AST from env non-responders and env responders to treatments. *P* values were calculated by unpaired nonparametric Mann-Whitney test.

Table.S1 Correlations between clinical parameters and the responsiveness of env-specific T cells to metabolic interventions.

clinical parameters	polyphenol		iACAT		MTA	
	R	P	R	P	R	P
age (year)	-0.026818	0.876619	-0.11442	0.506383	0.119441	0.487781
HBsAg (IU/mL)	0.110961	0.519404	0.238769	0.160785	0.047365	0.783844
HBcrAg (lgU/mL)	0.20296	0.235154	0.158895	0.354641	0.176324	0.303624
HBV DNA (IU/mL)	0.138681	0.419879	0.17112	0.318344	0.119995	0.485749
ALT (U/L)	-0.111512	0.51732	0.108929	0.527128	-0.017896	0.91749
AST (U/L)	-0.197041	0.256568	0.167929	0.33491	0.010657	0.951551
WBC (10^9/L)	-0.045239	0.799456	-0.09077	0.609676	-0.31143	0.072989
NEUT (%)	0.078111	0.660592	0.04203	0.813425	-0.034082	0.848248
NEUT (10^9/L)	0.004585	0.97947	0.014669	0.934378	-0.359539	0.036758
LYMPH (%)	-0.054715	0.758587	-0.083741	0.637746	-0.056006	0.753067
LYMPH (10^9/L)	-0.166208	0.347496	-0.209505	0.234372	-0.38967	0.022724
MONO (%)	-0.009252	0.958583	0.003593	0.983909	0.162691	0.357935
MONO (10^9/L)	-0.073768	0.678424	-0.060214	0.735152	-0.135654	0.444299
EO (%)	-0.326322	0.05963	-0.268604	0.124534	-0.323824	0.061724
EO (10^9/L)	-0.354041	0.039951	-0.297619	0.087364	-0.445126	0.008341
BASO (%)	-0.315708	0.068933	-0.04143	0.816041	-0.056587	0.750583
BASO (10^9/L)	-0.30427	0.080192	-0.043586	0.806644	-0.222879	0.205157
RBC (10^12/L)	-0.02629	0.88267	-0.040651	0.819442	-0.18293	0.300423
HGB (g/L)	0.065286	0.713746	0.033631	0.850233	-0.05381	0.762463
HCT (%)	0.006955	0.968862	0.068694	0.699477	-0.087186	0.623925
MCV (fL)	0.003363	0.984942	0.157485	0.373731	0.121647	0.493129
MCHC (g/L)	0.198391	0.26069	-0.141927	0.423317	0.067858	0.702968
MCH (pg)	-0.029225	0.869676	-0.136006	0.443109	0.062801	0.724207
RDW-CV (%)	-0.444649	0.00842	-0.149342	0.399242	-0.431492	0.010834
PLT (10^9/L)	0.05136	0.772988	0.213281	0.225853	-0.00833	0.96271
PRP	-0.214564	0.223008	-0.278555	0.110658	-0.19864	0.260079
PCT (%)	0.211484	0.237419	0.370769	0.033658	0.153537	0.39362
MPV (fL)	-0.208393	0.244505	-0.040784	0.82171	0.020266	0.910868
PDW (%)	-0.265697	0.135053	-0.03469	0.848013	-0.009697	0.957289
P-LCR (%)	-0.285499	0.107277	-0.040612	0.822451	-0.083062	0.645839
TP (g/L)	0.086065	0.623008	0.387759	0.021359	0.267706	0.119997
ALB (g/L)	0.093038	0.595013	0.15914	0.361173	-0.006343	0.971154
GLB (g/L)	0.0131	0.940463	0.311061	0.068935	0.254775	0.139669
A/G	0.036435	0.835391	-0.192812	0.267122	-0.20737	0.231966
PA (mg/L)	-0.060254	0.730973	-0.200364	0.248471	-0.192644	0.267545
D-bil (umol/L)	-0.129222	0.4594	-0.111975	0.521903	0.090667	0.604471
T-bil (umol/L)	-0.195741	0.259783	-0.10732	0.539457	0.019473	0.911591
D/T	0.182157	0.294955	0.051841	0.767418	0.200043	0.249246
AST/ALT	0.045811	0.793848	0.170134	0.32851	0.165429	0.342257

**ALP (U/L)**	0.038645	0.825555	0.143068	0.412278	0.346704	0.041309
**r-GGT (U/L)**	-0.091706	0.600319	0.004627	0.978954	0.035471	0.839687
**TBA (umol/L)**	-0.124535	0.475978	-0.066597	0.703864	-0.045029	0.797292
**CHE (U/L)**	-0.062338	0.722028	-0.077182	0.659439	-0.055746	0.750433
**LDH-L (U/L)**	-0.311874	0.068174	-0.068437	0.69607	-0.228479	0.186784
**LAP (U/L)**	-0.159412	0.478563	0.146934	0.514072	0.013559	0.952244
**ADA (U/L)**	-0.037262	0.831707	0.046275	0.791807	0.093811	0.591944
**a-AMY (U/L)**	0.012161	0.957166	-0.112808	0.617195	-0.078576	0.728158
**CK (U/L)**	-0.08831	0.695946	-0.060555	0.788936	-0.261952	0.238936
**UREA (mmol/L)**	-0.122608	0.482886	-0.420581	0.011879	-0.295017	0.085345
**CREA (umol/L)**	-0.013814	0.937224	-0.094587	0.588869	-0.121433	0.487121
**EGFR**	-0.032905	0.85115	0.057177	0.744238	-0.181831	0.295835
**UA (umol/L)**	-0.016674	0.924262	-0.090928	0.603426	-0.016741	0.923955
**TC (mmol/L)**	-0.198118	0.331953	0.233493	0.250974	-0.08312	0.686445
**TG (mmol/L)**	-0.140438	0.493798	-0.02326	0.910202	0.151163	0.461046
**HDL-C (mmol/L)**	0.093472	0.671415	0.121632	0.580363	-0.35393	0.097547
**LDL-C (mmol/L)**	0.152072	0.488506	0.394596	0.062418	0.290605	0.178556
**ApoA1 (g/L)**	0.026967	0.90279	0.072224	0.743302	-0.490604	0.017461
**ApoB (g/L)**	0.063567	0.773235	0.30366	0.158951	0.335229	0.117891
**Lp (a) (mg/L)**	0.142398	0.516889	0.275334	0.203528	0.170991	0.435342
**Weight (kg)**	-0.091678	0.692675	0.113747	0.623472	0.024691	0.915394
**Height (m)**	-0.086617	0.708906	0.21875	0.34076	0.173772	0.451257
**BMI**	-0.052632	0.820753	0.117571	0.611768	0.124675	0.590261
